# Clinical and Radiographic Factors Affecting the Outcomes of Decompression Surgery in Clinical and Radiographic Degenerative Spondylolisthesis (CARDS) Classification Type C Degenerative Lumbar Spondylolisthesis

**DOI:** 10.7759/cureus.106456

**Published:** 2026-04-05

**Authors:** Atsushi Kawano, Kiyoshi Tarukado, Kazuya Yokota, Kazu Kobayakawa, Hirokazu Saiwai, Kenichi Kawaguchi, Yasuharu Nakashima

**Affiliations:** 1 Orthopaedic Surgery, Kyushu University Hospital, Fukuoka, JPN; 2 Orthopaedic Surgery, Graduate School of Medical Sciences, Kyushu University, Fukuoka, JPN

**Keywords:** decompression surgery, degenerative spondylolisthesis, lumbar spine, pelvic tilt, spinal instability

## Abstract

Introduction

The optimal surgical strategy for Clinical and Radiographic Degenerative Spondylolisthesis (CARDS) Type C remains controversial, particularly regarding decompression alone versus fusion surgery. Evidence focusing on outcomes after decompression alone in this subgroup is limited. This study evaluated clinical outcomes after decompression in patients with L4 degenerative spondylolisthesis classified as CARDS Type C and identified preoperative factors associated with postoperative walking function.

Methods

This retrospective analysis of prospectively collected data included 59 patients who underwent decompression surgery for lumbar spinal stenosis associated with L4 degenerative spondylolisthesis between April 2018 and March 2022 and were followed for at least one year. Thirty-four patients classified as CARDS Type C were analyzed. Clinical outcomes were assessed using visual analog scale scores for low back pain and leg pain, the Oswestry Disability Index, and the Japanese Orthopaedic Association Back Pain Evaluation Questionnaire (JOABPEQ) preoperatively and at six months and one year postoperatively. Preoperative radiographic parameters, including slip distance, segmental motion, and spinopelvic alignment, were evaluated. Factors associated with changes in JOABPEQ walking function were analyzed using correlation analysis, stepwise multiple regression, and receiver operating characteristic (ROC) curve analysis.

Results

All clinical outcome measures showed significant improvements at six months and one year after surgery. Stepwise multiple regression identified two independent factors associated with poorer improvement in walking function: preoperative change in L4 slip distance and preoperative pelvic tilt (PT). ROC analysis for treatment effectiveness based on the JOABPEQ walking function score showed areas under the curve of 0.65 for slip distance change and 0.76 for PT, with optimal cutoff values of 4 mm and 30 degrees, respectively.

Conclusions

Decompression surgery provides generally favorable outcomes in CARDS Type C. However, postoperative improvement in walking function may vary depending on preoperative radiographic characteristics. Greater dynamic instability and increased PT were associated with limited functional recovery. These findings suggest that decompression alone may achieve favorable outcomes in selected patients with less dynamic instability and lower PT.

## Introduction

Lumbar degenerative spondylolisthesis is a common cause of spinal stenosis, and the treatment option between decompression alone and decompression with fusion remains controversial. One major reason for this controversy is the concept of spinal instability. Instability is often evaluated using multiple radiographic parameters, and the assessment is inconsistent and subjective, which leads to wide variation in surgical decision-making [1].

Several studies have suggested that decompression alone may be appropriate when the slip distance is less than 5 mm [2], and this criterion is commonly used in clinical practice. However, treatment strategies for degenerative spondylolisthesis are still not standardized. One possible reason is the lack of a classification system that directly links disease characteristics to surgical decision-making.

The Clinical and Radiographic Degenerative Spondylolisthesis (CARDS) classification was proposed by Kepler and colleagues in 2015 as a common language for degenerative spondylolisthesis [3]. The CARDS classification divides the disease into four types: Type A, with complete disc space collapse and vertebral body contact; Type B, with slip less than 5 mm; Type C, with slip of 5 mm or more; and Type D, characterized by posterior disc opening with kyphotic angulation at the slip level observed on at least one lateral radiographic view (neutral, flexion, or extension). This classification has been reported to be useful for surgical planning and outcome prediction [4,5]. Previous studies have shown that in CARDS Type D, fusion surgery provides better outcomes than decompression alone [6] and that Type D patients achieve greater clinical improvement after posterior interbody fusion compared with other CARDS types [7]. However, the appropriate surgical strategy for CARDS Type C remains unclear. Specifically, it is not well understood to what extent decompression alone can provide satisfactory outcomes in patients with a slip of 5 mm or more.

The first purpose of this study was to evaluate the clinical outcomes of decompression surgery in patients with L4 degenerative spondylolisthesis classified as CARDS Type C, defined as a slip of 5 mm or more. The second purpose was to identify preoperative factors associated with postoperative functional improvement that may help guide surgical decision-making.

## Materials and methods

This was a retrospective analysis of prospectively collected data. Between April 2018 and March 2022, 59 patients with lumbar spinal stenosis associated with L4 degenerative spondylolisthesis underwent decompression surgery and were followed for at least one year. All patients were classified into four groups according to the preoperative CARDS classification using standing radiographs, with additional assessment based on dynamic flexion and extension lateral radiographs [5]. Among these patients, 34 (57.6%) classified as CARDS Type C were included in this study.

During the study period, decompression alone was indicated for patients with cauda equina syndrome caused by central canal stenosis, mixed-type neurological symptoms, or L5 nerve root symptoms within the spinal canal. Patients with L4 radiculopathy caused by L4-5 foraminal stenosis concomitant with L4 degenerative spondylolisthesis underwent posterior interbody fusion using the transforaminal lumbar interbody fusion (TLIF) technique. For patients who required decompression at a single level of L4/5, microendoscopic laminectomy was performed. For patients who required decompression at two or more levels, including L4/5, spinous process-splitting laminectomy was selected. During the study period, fusion surgery was not performed in any patients classified as CARDS Type C. Accordingly, all patients with CARDS Type C included in this study were treated with decompression surgery alone.

Clinical outcomes were evaluated using the visual analog scale (VAS) [8] for low back pain (LBP), the VAS for leg pain, the Oswestry Disability Index (ODI) [9], and the Japanese Orthopaedic Association Back Pain Evaluation Questionnaire (JOABPEQ) [10]. These assessments were performed preoperatively and at six months and one year after surgery.

**Figure 1:**
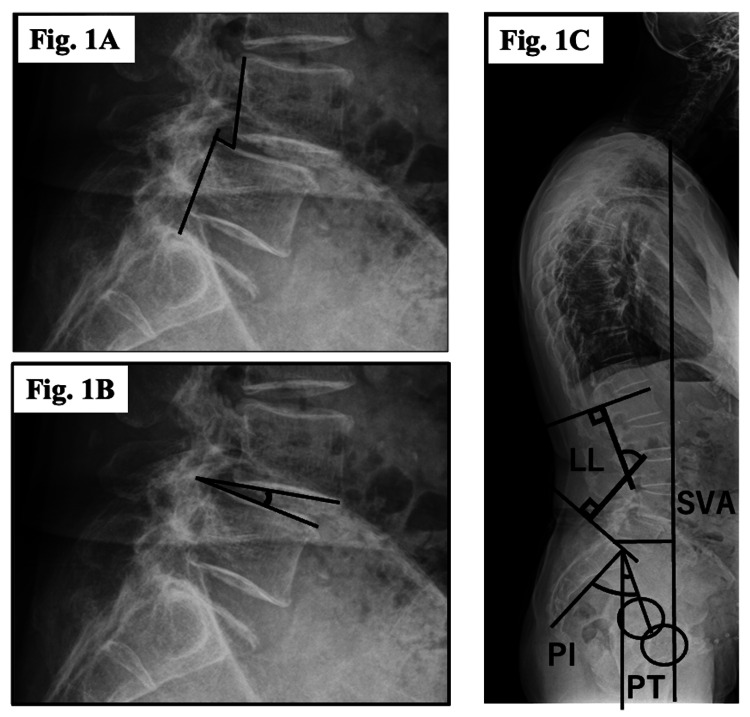
Radiographic measurements. The radiographic images were obtained from patients treated at our institution, and the illustrations were created by the authors. A: On lateral lumbar radiographs, the slip distance was measured from the posterior vertebral margin in the neutral, extension, and flexion positions. The difference in slip distance between extension and flexion was defined as the change in slip distance. B: On lateral lumbar radiographs, the segmental angle was measured between the inferior endplate of the upper vertebra and the superior endplate of the lower vertebra. The difference between extension and flexion was defined as the change in segmental motion. C: On whole-spine standing lateral radiographs, the sagittal vertical axis (SVA) was defined as the horizontal distance from the posterior superior corner of the sacrum to the vertical plumb line dropped from the center of the C7 vertebral body. Lumbar lordosis (LL) was measured as the angle between the superior endplates of L1 and S1. Pelvic incidence (PI) was defined as the angle between the line connecting the midpoint of the sacral endplate to the bicenter of the femoral heads and the perpendicular to the sacral endplate. Pelvic tilt (PT) was defined as the angle between the line connecting the midpoint of the sacral endplate to the bicenter of the femoral heads and the vertical line.

Radiographic evaluation was performed using plain radiographs. Preoperative measurements included L4 slip distance in extension and flexion positions, with the change in slip distance defined as instability; L4/5 segmental range of motion (ROM) in extension and flexion, with the change in angle defined as segmental ROM; and global spinal alignment parameters, including sagittal vertical axis (SVA), pelvic tilt (PT), pelvic incidence (PI), lumbar lordosis (LL), and PI minus LL (PI-LL) (Figure 1).

Statistical analyses were performed using the t-test, Spearman’s rank correlation coefficient, multiple regression analysis with a stepwise method, and nominal logistic regression analysis. A p-value of less than 0.05 was considered statistically significant. Statistical analyses were performed using JMP® Student Edition version 18.2.2 (SAS Institute Inc., Cary, NC, USA).

## Results

A total of 59 patients were classified according to the CARDS classification as follows: Type A, one patient (1.7%); Type B, five patients (8.5%); Type C, 34 patients (57.6%); and Type D, 19 patients (32.2%). Subsequent analyses in this study focused on patients classified as CARDS Type C (n = 34, 57.6%). Baseline characteristics of patients with CARDS Type C, including patient demographics, clinical factors, and radiographic parameters, are summarized in Table 1.

**Table 1:** Baseline characteristics of CARDS Type C patients Data ± SE JOABPEQ: Japanese Orthopaedic Association Back Pain Evaluation Questionnaire

Variable	Values
Age	72.9 ± 1.8
Gender (M:F)	18:16
Preoperative Oswestry Disability Index (ODI) (%)	47.0 ± 2.7
Preoperative JOABPEQ pain-related disorders	45.3 ± 5.1
Preoperative JOABPEQ lumbar function	47.2 ± 4.8
Preoperative JOABPEQ walking ability	24.6 ± 4.1
Preoperative JOABPEQ social life condition	32.1 ± 3.6
Preoperative JOABPEQ mental health	40.6 ± 3.0
Preoperative low back pain (LBP)/leg pain visual analog scale (VAS)	53.9 ± 4.7/68.6 ± 3.4
Change in slip distance	1.0 ± 0.7
Segmental range of motion (ROM)	4.7 ± 0.6
Pelvic tilt (PT)	26.1 ± 1.4
Pelvic incidence (PI)	56.9 ± 1.9
Lumbar lordosis (LL)	41.6 ± 2.2
Sagittal vertical axis (SVA)	35.9 ± 7.5
Surgical procedures (Microendoscopic laminectomy (MEL):laminectomy)	25:9
Treatment effective rate (JOABPEQ walking function) (n, %）	24, 70.6%

No significant differences were observed between surgical procedures (microendoscopic laminectomy and spinous process-splitting laminectomy) in any of the evaluated demographic, clinical, or radiographic variables (Table 2).

**Table 2:** Comparison between surgical procedures Data ± SE ODI: Oswestry Disability Index; JOABPEQ: Japanese Orthopaedic Association Back Pain Evaluation Questionnaire; PT: pelvic tilt

Variable	MEL (n=25)	Laminectomy (n=9)	P-value
Age	72.5 ± 1.3	74.2 ± 1.7	0.43
Gender (M:F)	12:13	6:3	0.34
Preoperative ODI (%)	46.0 ± 2.7	49.9 ± 7.4	0.63
Preoperative JOABPEQ walking ability	28.0 ± 5.1	15.1 ± 6.2	0.12
Change in slip distance	0.56 ± 0.88	2.3 ± 1.3	0.28
PT	26.4 ± 1.8	25.3 ± 2.2	0.70
Postoperative JOABPEQ walking ability	54.9 ± 6.2	56.4 ± 12.0	0.91
Treatment effective rate (JOABPEQ walking function) (n, %）	16, 64%	8, 88.9%	0.16

**Figure 2:**
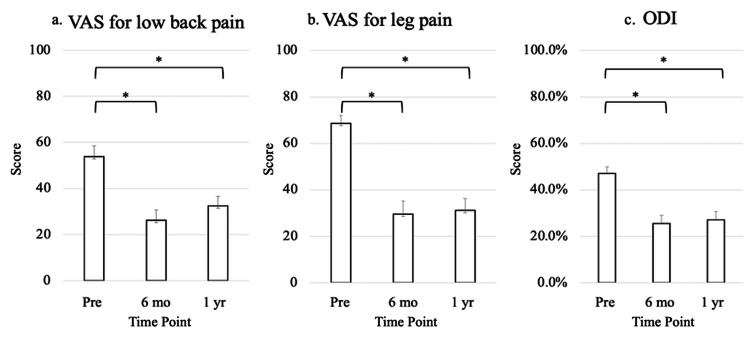
Postoperative Change in VAS Scores and ODI. (a) Visual analog scale for low back pain; (b) Visual analog scale for leg pain; and (c) Oswestry Disability Index. All parameters showed significant improvement at six months and one year after surgery compared with preoperative values. An asterisk (*) indicates a statistically significant difference (p < 0.05).

The VAS scores for LBP and leg pain, as well as the ODI, showed significant improvement at six months and one year after surgery compared with preoperative values (Figure 2).

**Figure 3:**
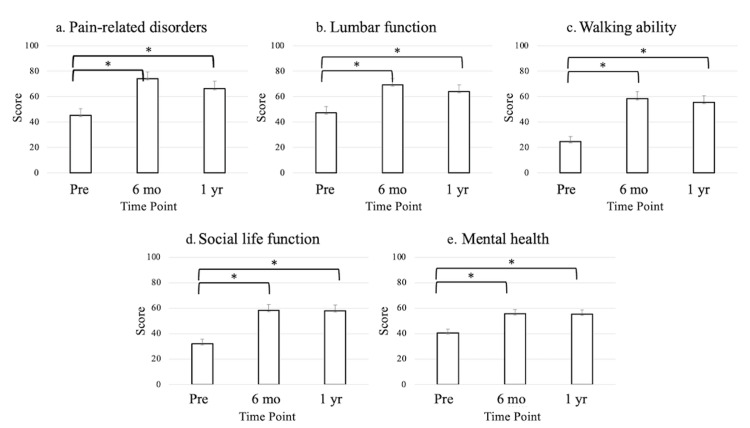
Postoperative changes in each domain of the JOABPEQ. (a) Pain-related disorders; (b) Lumbar function; (c) Walking ability; (d) Social life function; and (e) Mental health. All domains showed significant improvement at six months and one year after surgery. An asterisk (*) indicates a statistically significant difference (p < 0.05).

Similarly, all domains of the JOABPEQ demonstrated significant improvement at both postoperative time points (Figure 3).

Given that improvement of intermittent claudication is a major goal of surgery, walking ability was examined to identify factors associated with poor postoperative outcomes. Treatment effectiveness was defined as a postoperative increase of 20 points or more in the JOABPEQ walking function score or a postoperative score of 90 points or higher [11]. Based on this definition, patients were classified into effective and ineffective groups, and preoperative variables were compared between the groups. Preoperative PT was significantly higher in the ineffective group (Table 3).

**Table 3:** Comparison between patients with and without treatment effectiveness in JOABPEQ walking function Data ± SE ODI: Oswestry Disability Index; JOABPEQ: Japanese Orthopaedic Association Back Pain Evaluation Questionnaire; VAS: visual analog scale; LBP: low back pain; PT: pelvic tilt; PI: pelvic incidence; LL: lumbar lordosis; SVA: sagittal vertical axis

Variable	Effective group	Ineffective group	P-value
Age	72.5 ± 1.4	74.0 ± 1.5	0.47
Gender (M:F)	14:10	4:6	0.33
Preoperative ODI (%)	48.5 ± 3.6	43.4 ± 3.6	0.32
Preoperative leg pain VAS	70.4 ± 4.2	64.3 ± 5.4	0.38
Preoperative LBP VAS	50.9 ± 5.8	60.9 ± 7.7	0.31
Preoperative L4 slip distance (flexion)	8.0 ± 0.6	10.1 ± 1.0	0.09
Preoperative L4 slip distance (extension)	7.7 ± 0.7	7.2 ± 1.0	0.73
Preoperative change in L4 slip distance	0.38 ± 0.8	2.6 ± 1.5	0.20
Preoperative change in L4/5 segmental angle	5.3 ± 0.8	3.1 ± 0.9	0.07
PT	23.8 ± 1.6	31.3 ± 2.4	0.02
PI	54.6 ± 1.8	62.3 ± 4.2	0.12
LL	43.0 ± 2.8	38.5 ± 3.5	0.33
SVA	27.8 ± 7.8	54.6 ± 16.0	0.16

Correlation analysis between preoperative variables and the change in the JOABPEQ walking function score over one year revealed significant correlations for four factors: preoperative L4 slip distance in flexion, preoperative change in L4 slip distance, preoperative PT, and preoperative PI-LL (Table 4).

**Table 4:** Preoperative factors associated with changes in JOABPEQ walking function at one year *SVA: sagittal vertical axis. **PT: pelvic tilt. ***PI: pelvic incidence. ****LL: lumbar lordosis. Correlation analysis between each preoperative factor and the one-year change in JOABPEQ walking function identified four factors with significant correlations. JOABPEQ: Japanese Orthopaedic Association Back Pain Evaluation Questionnaire; LBP: low back pain; PT: pelvic tilt; PI: pelvic incidence; LL: lumbar lordosis; SVA: sagittal vertical axis

Preoperative factors	Spearman’s rank correlation coefficient	P-value
Preoperative L4 slip distance (flexion)	-0.3650	0.0367
Preoperative L4 slip distance (extension)	0.0372	0.8374
Preoperative change in L4 slip distance	-0.3541	0.0399
Preoperative L4/5 segmental angle (flexion)	0.0079	0.9652
Preoperative L4/5 segmental angle (extension)	0.2494	0.1616
Preoperative change in L4/5 segmental angle	0.3191	0.0658
Preoperative SVA*	-0.2029	0.2575
Preoperative PT**	-0.4185	0.0154
Preoperative PI***	-0.1800	0.3163
Preoperative LL****	0.1979	0.2695
Preoperative PI–LL	-0.4164	0.0143

These four factors were entered into a stepwise multiple regression analysis. The analysis identified two independent preoperative factors significantly associated with poorer improvement in walking function: greater preoperative change in L4 slip distance, representing dynamic instability, and higher preoperative PT (Table 5).

**Table 5:** Preoperative predictors of one-year changes in JOABPEQ walking function Greater change in slip distance on dynamic radiographs and higher preoperative PT were independently associated with poorer improvement in walking function. PT: pelvic tilt; JOABPEQ: Japanese Orthopaedic Association Back Pain Evaluation Questionnaire

Preoperative factors	P-value	Standardized β
Preoperative change in L4 slip distance	0.0100	-0.401
Preoperative PT	0.0018	-0.499

**Figure 4:**
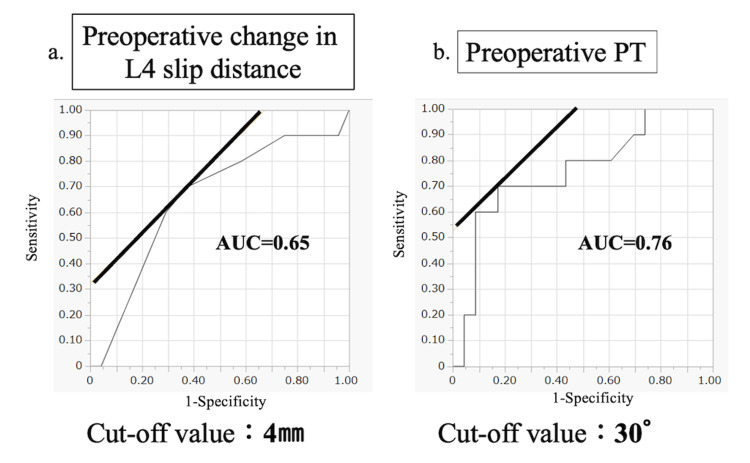
Receiver operating characteristic (ROC) analysis of preoperative instability and pelvic tilt for predicting treatment effectiveness based on the JOABPEQ. Treatment effectiveness was defined as an improvement of ≥20 points or a postoperative score of ≥90 points in the JOABPEQ walking function domain. (a) Preoperative change in the L4 slip distance. The area under the curve (AUC) was 0.65, indicating moderate discriminative ability. The optimal cutoff value was 4 mm. (b) Preoperative pelvic tilt (PT). The AUC was 0.76, indicating good discriminative ability. The optimal cutoff value was 30 degrees. JOABPEQ: Japanese Orthopaedic Association Back Pain Evaluation Questionnaire

A nominal logistic regression analysis was performed using the two significant preoperative factors as predictors. Receiver operating characteristic curve analysis for treatment effectiveness based on the JOABPEQ walking function score showed that the area under the curve (AUC) was 0.65 for preoperative change in L4 slip distance and 0.76 for preoperative PT. The optimal cutoff values were 4 mm for the change in L4 slip distance and 30 degrees for PT (Figure 4).

## Discussion

This study provides clinically meaningful insights into surgical decision-making for CARDS Type C degenerative spondylolisthesis, a subgroup for which the optimal treatment strategy remains controversial. Overall, decompression surgery resulted in significant improvement in pain- and disability-related outcomes at one year postoperatively, including LBP, leg pain, the ODI, and all domains of the JOABPEQ. However, improvement in walking function was not uniform across patients. Greater preoperative change in slip distance on dynamic radiographs, reflecting increased instability, was associated with limited improvement in walking function, with a cutoff value of 4 mm. In addition, increased preoperative PT, a marker of sagittal compensatory imbalance, was also associated with poorer walking function outcomes, with a cutoff value of 30 degrees. Together, these findings highlight specific radiographic factors that may help identify CARDS Type C patients who are less likely to achieve optimal functional recovery with decompression surgery alone.

In this study, the mean improvement in the JOABPEQ walking function score after decompression surgery was 30 points. In contrast, a previous study reported a mean improvement of 43 points in walking function after lumbar fusion surgery for degenerative spondylolisthesis [12]. Improvements in the other JOABPEQ domains appeared comparable to those reported by Hiyama et al. [12], although the data are not shown. This difference suggests that the degree of improvement in walking function may vary depending on the surgical procedure and that fusion surgery may provide superior outcomes to decompression alone in selected patients. Based on these findings, we further examined factors that limit improvements in walking function. CARDS Type C is defined as spondylolisthesis with a slip of 5 mm or more, and our results indicate that patients with additional dynamic instability of 4 mm or more experience limited benefit from decompression alone. This finding is consistent with a report by Hipp and colleagues [13], who showed poorer outcomes in patients with large slip changes between flexion and extension radiographs. Taken together, these results suggest that adding fusion surgery may be considered in CARDS Type C patients with significant dynamic instability (4 mm or more). Although the predictive ability of preoperative change in slip distance was modest, with an AUC of 0.65, this finding suggests an acceptable level of discrimination rather than a definitive threshold. Therefore, the 4 mm cutoff value should not be interpreted as an absolute indication for fusion surgery but rather as a supportive parameter to assist surgical decision-making in combination with clinical findings and other radiographic factors.

We also found that greater preoperative PT was associated with poorer improvements in walking function. Preoperative PT demonstrated a higher predictive ability, with an AUC of 0.76, suggesting good discriminative performance. PT reflects compensatory pelvic retroversion during standing and walking and is therefore closely related to functional impairment, which may explain its stronger association with postoperative improvements in walking function compared with static alignment parameters such as PI-LL. Accordingly, preoperative PT may serve as a more reliable indicator when considering the potential need for fusion surgery in CARDS Type C patients. Previous studies have reported a relationship between PT and walking ability, showing that patients with increased PT have difficulty walking comfortably [14,15]. Posterior interbody fusion techniques such as TLIF have been reported to restore LL [16,17]. Previous studies have also demonstrated that restoration of LL can reduce pelvic retroversion and improve PT by correcting spinopelvic mismatch, thereby decreasing the need for compensatory mechanisms during standing and walking [18,19]. From a biomechanical perspective, an improvement in LL leads to better pelvic alignment, which may secondarily result in a reduction in PT and improvement in functional outcomes. Although the present study did not directly evaluate postoperative changes in PT, these findings suggest that patients with a preoperative PT greater than 30 degrees may benefit from interbody fusion surgery aimed at restoring LL, which may contribute to improved spinopelvic balance and walking function. Further prospective studies are needed to clarify the relationship between LL restoration, PT correction, and functional recovery in CARDS Type C patients.

This study has several limitations. It was a single-center study with a relatively small sample size, which may introduce selection bias. Larger multicenter studies are needed to confirm our findings. In addition, although the inclusion of clinical variables such as age and baseline functional status would have strengthened the analysis, the limited sample size restricted the number of variables that could be included in the multivariate model. Therefore, additional variables were not incorporated to avoid the risk of overfitting. In addition, this study does not include a comparison group undergoing fusion surgery, as no CARDS Type C patients underwent fusion during the study period. The absence of a control group is a major limitation of this study, and therefore, our findings cannot be used to directly compare decompression and fusion or to determine the superiority of one surgical strategy over the other. Consequently, it remains unclear whether interbody fusion surgery can reliably restore LL or improve sagittal alignment in CARDS Type C patients. Prospective studies are required to determine whether fusion surgery improves sagittal alignment and clinical outcomes in this patient population.

## Conclusions

Decompression alone generally provides favorable clinical outcomes in patients with CARDS Type C degenerative spondylolisthesis. However, improvement in walking function may vary depending on preoperative radiographic characteristics. Greater dynamic instability and increased PT were associated with limited functional recovery, suggesting that spinopelvic alignment and segmental instability should be carefully evaluated during surgical planning. Importantly, these findings indicate that even in CARDS Type C, decompression alone may achieve favorable outcomes in selected patients with less dynamic instability and lower PT.
